# The Microencapsulation of Maqui (*Aristotelia chilensis* (Mol.) Stuntz) Juice by Spray-Drying and Freeze-Drying Produces Powders with Similar Anthocyanin Stability and Bioaccessibility

**DOI:** 10.3390/molecules23051227

**Published:** 2018-05-20

**Authors:** Carolina Fredes, Camila Becerra, Javier Parada, Paz Robert

**Affiliations:** 1 Departamento de Ciencia de los Alimentos y Tecnología Química, Facultad de Ciencias Químicas y Farmacéuticas, Universidad de Chile, Sergio Livingstone Pohlhammer 1007, Independencia 8380492, Chile; carolina.fredes@postqyf.uchile.cl (C.F.); camilaj.bc@gmail.com (C.B.); 2 Institute of Food Science and Technology, Faculty of Agricultural Sciences, Austral University of Chile, Valdivia 5090000, Chile; javier.parada@uach.cl

**Keywords:** encapsulation methods, in vitro digestion, maltodextrin, soy protein isolate, yogurt

## Abstract

The microencapsulation of maqui juice by spray-drying and freeze-drying was studied as a strategy to protect anthocyanins in new food formulations in order to improve the anthocyanin retention before consumption and the bioaccessibility. It is well known that the encapsulation method affects both the shape and size of powders, being assumed that undefined forms of freeze-drying powders might affect their stability due to the high permeability to oxygen. The objective of this study was to compare the microencapsulation of maqui juice by spray-drying and freeze-drying, evaluating the stability of specific anthocyanins in yogurt and after in vitro digestion. Results indicated that most relevant differences between spray-drying and freeze-drying powders were the morphology and particle size that affect their solubility (70.4–59.5%) when they were reconstituted in water. Nevertheless these differences did not affect the stability of anthocyanins as other research have proposed. Both encapsulation methods generated powders with a high stability of 3-*O*-monoglycosylated anthocyanins in yogurt (half-life values of 75–69 days for delphinidin-3-sambubioside). Furthermore, no significant differences in the bioaccessibility of anthocyanins between maqui juice powders (44.1–43.8%) were found. In conclusion, the microencapsulation of maqui juice by freeze-drying is as effective as spray-drying to produce new value-added food formulations with stable anthocyanins.

## 1. Introduction

Maqui (*Aristotelia chilensis* (Mol.) Stuntz, Elaeocarpaceae) is a native berry from Chile, recognized worldwide as a rich source of health-promoting anthocyanins with antioxidant, anti-inflammatory, anti-diabetic, and hypoglycaemic activities [1]. New maqui fruit-based products appear frequently on the international market correlating with an increase in Chilean exports of maqui fruit (frozen, juice, dehydrated, canned, and other fruit preparations) worth US $308,562 in 2017, with a mean value of US $4.3–6.5/kg [2]. While the potential health properties of maqui fruit are well known, the stability of anthocyanins in maqui products currently on the market has been scarcely studied. For this reason, it is necessary to study microencapsulation methods to develop new value-added maqui fruit foods with stable anthocyanins that convey health benefits to consumers.

The anthocyanin content of maqui is significantly higher than in other berry-type fruits [3]; where eight anthocyanins (70% delphinidin and 30% cyanidin glucosides) have been identified in the anthocyanin profile of the maqui fruit [4]. However, anthocyanins in general are susceptible to degradation when they are exposed to environmental conditions such as heat, oxygen, and changes in pH, among others [5]. Therefore, encapsulation technology can be used as a strategy to protect maqui anthocyanins in value-added products such as health food ingredients.

Spray-drying (SD) is the most common technique used to encapsulate anthocyanins [5,6]. It is useful for encapsulating heat-sensitive materials because of its short drying times (5–30 s) [7]. Moreover, spray dryers are commonly available in the food and pharmaceutical industries. However, during the last 10 years, freeze-drying (FD) has become more widespread in the food industry [6]. Freeze-drying technique is based on the removal of water from a frozen product by sublimation and has been used as an alternative method to encapsulate anthocyanins [8]. Although at least two previous studies have compared SD and FD as methods to encapsulate anthocyanins from black glutinous rice bran [8] and blueberries [9], a comparison of the stability of encapsulated maqui anthocyanins in food matrices and the gastrointestinal (GI) tract using these two methods has not been evaluated. 

Anthocyanin encapsulation has been studied as protection method against environmental conditions during storage, in order to extend shelf life. However, to develop more effective health food ingredients, the anthocyanin stability in food matrices and simulated GI tracts should be assessed [6]. In this context, the selection of the encapsulation method plays an important role in the powder properties that would influence the anthocyanin retention before consumption and the bioaccessibility (BA). For example, Laokuldilok and Kanha [8] indicated that the morphology of FD powders might affect their stability during storage due to the high permeability to oxygen. Maltodextrins (MD) of different dextrose equivalents are biopolymers widely used as encapsulating agent (EA) in the encapsulation of anthocyanins, because MD have low viscosity at high solid content, bland flavor and colourless solutions [6,7]. MD is water soluble and when it is added to liquid foods (as yogurt) the active compounds are released and they are exposed to food conditions. In this study, maqui-juice microparticles were designed with maltodextrin (MD) blend with soy protein isolate (SPI), since SPI is partially soluble, which would lead to a decrease in the solubility of the maqui-juice microparticles. Thus, the objective of this study was to evaluate two different encapsulation methods, spray-drying and freeze-drying, regarding stability of each specific anthocyanin in yogurt and their bioaccessibility in vitro.

## 2. Results and Discussion

### 2.1. The Encapsulation of Anthocyanins from Maqui Juice (MJ) by SD and FD

In order to find the optimal conditions for encapsulation by SD, a response surface methodology (RSM) experiment was carried out. The experimental data are shown in Table 1. Equations were fitted to predict yield, recovery, and encapsulation efficiency (EE) of total anthocyanins (TA) and specific anthocyanins. Table 2 shows the predictive models following the general equation:

(1) $$y_{n}=\beta_{n0}+\beta_{n1}x_{1}+\beta_{n2}x_{2}+\beta_{n3}x_{1}^{2}+\beta_{n4}x_{1}x_{2}+\beta_{n5}x_{2}^{2}$$

where $\beta_{n}$ are the regression coefficients for each response variable $y_{n}$, $x_{1}$ is the inlet air temperature, and $x_{2}$ the MJ/maltodextrin (MD)-soy protein isolate (SPI) ratio.

The coefficients of determination (R^2^) and R^2^-adjusted for the predictive models were higher than 0.90 and 0.82, respectively, for each response variable except for recovery (0.42 and 0.0, respectively), suggesting that the predictive models seemed to reasonably represent the observed values, with the exception of recovery. Therefore, 8 of the 9 responses could be sufficiently explained by the models, while for recovery, results suggest that its optimal condition is outside the experimental zone. Anthocyanin recovery reveals the effect of the drying process on anthocyanin content. In this study, high recovery results (≥82.6%) indicate that the temperatures used in SD did not have a great effect on the degradation of anthocyanins.

Multiple response optimization methodology was performed in order to obtain optimal conditions that maximize all variables. These optimal conditions were an inlet air temperature of 186 °C and a MJ/MD-SPI ratio of 1:5. The corresponding predictive values were a yield of 73.4%, a recovery of 93.5%, and an EE of TA of 90.9%. The predictive yield was relatively low but in line with previous reports [6], whereas recovery (93.5%) and anthocyanin EE (>87%) were higher than expected from previous research [10]. In order to compare the properties of SD powders obtained under optimal conditions with FD powders, a MJ/MD-SPI ratio of 1:5 was used to formulate the FD powders.

### 2.2. Characterization of the MJ Powders Obtained by SD and FD

The anthocyanin profile of MJ was similar than those previously described [4,11,12,13,14,15], showing eight anthocyanins (del-3-sa-5-glu, del-3,5-diglu, cy-3,5-diglu+cy-3-sa-5-glu (coeluted in peak 3–4), del-3-sa, del-3-glu, cy-3-sa and cy-3-glu). Additionally, cy-3-sa was found under the limit of quantification (0.16 µg/mL). The anthocyanin profiles for MJ powders (SD and FD) were similar to MJ where 3,5-*O*-diglycosylated anthocyanins (≈70%) predominated over 3-*O*-glycosylated anthocyanins (≈30%) in both MJ and the MJ powders (Table 3). In concordance with the high anthocyanin content of MJ (17.5 mg/g) as a raw material, both MJ powders showed high anthocyanin content (SD, 3.3 mg/g and FD, 3.3 mg/g) in comparison to microparticles in powder from other raw materials such as Andes berry (0.1 mg/g) [16] and bayberry (0.6 mg/g) [17].

The EE has been used to determine if the anthocyanins were encapsulated. EE represents anthocyanin-biopolymer interaction due to electrostatic interactions, hydrogen bonding, hydrophobic interactions, or Van der Waals forces [7]. However, encapsulation by spray-drying is considered an immobilization technology rather than a true encapsulation technology because some active compounds remain exposed on the microparticle surface [6]. Thus, the EE is a way to gauge successful anthocyanin encapsulation which should result in a powder that has minimum surface anthocyanin content on the microparticles and maximum retention of active compounds [5]. According to our results, and in line with the predictive value for SD, the EE of TA was high for SD (92.5%) and FD (>93.0%) powders (Table 4) where the encapsulation method did not significantly affect the EE. Similar results showing high EE of TA were described by Santana et al. [18] for jussara microparticles (88.3–99.3%) using a mixture of arabic gum/modified starch/SPI and SD. Furthermore, our results showed that the mixture of polysaccharides and proteins improved the EE of TA, as compared to using either MD (58.5%) or SPI (86.6%) alone, in the microencapsulation of pomegranate juice using SD [10]. In line with the EE of TA results, the EE of specific anthocyanins did not show significant differences between SD and FD methods, although the EE of 3,5-*O*-diglycosylated anthocyanins was significantly higher than EE of 3-*O*-monoglycosylated anthocyanins (Table 4). This suggests a better interaction between the EA (MD-SPI) and 3,5-*O*-diglycosylated anthocyanins by hydrogen bonding, which could be attributed to the greatest number of hydroxyl groups for 3,5-*O*-diglycosylated anthocyanins.

The recovery of anthocyanins in the SD powders (99.8%) was in line with the predictive value and significantly higher than the FD powders (91.8–91.9%) (Table 4). However, these values were higher than those reported by Laokuldilok and Kanha [8] for black glutinous rice bran microparticles using SD (47.7%) and FD (71.9%).

The SD yield (64.1%) was 10% lower than the predictive value and significantly lower than FD (94.6%). Similarly, Laokuldilok and Kanha [8] reported differences in the yield between SD (>64%) and FD (85%). This can be explained by the high viscosity of the feed solution when SPI is used as an EA, which causes more solids to stick to the wall of the dryer chamber, resulting in less powder at the end of the SD process.

The physical characteristics (Table 4) showed that the water activity (a_w_) of SD powders was significantly higher than FD powders, but all of the values were low (<0.3), therefore avoiding potential microbial food spoilage [16]. Additionally, the moisture content results were in the range acceptable for food powders (3–10%) but they were also significantly higher in SD powders than FD powders. In concordance with a high moisture content, SD powders had significantly lower hygroscopicity and higher bulk density than FD powders. Moreover, it is important to notice that no significant differences between FD powders obtained by grinded with coffee grinder (FDc) and porcelain mortar (FDm) for any of these physical parameters were found (Table 4), reflecting that the grinding process did not affect a_w_, moisture content, hygroscopicity, nor the bulk density of the two types of FD powders.

The ability of the powders to reconstitute in water is significant for the development of food ingredients. As expected, including SPI in the MJ powders decreased solubility in comparison to using only MD [18]. The solubility of SD powders (70.4%) was significantly higher than FD powders (59.9–59.1%), which may be attributed to differences in the morphology of powders and the corresponding contact surface with water. In line with physical parameters, no significant difference in solubility between FDc and FDm were found. Furthermore, the solubility results may also be attributed to the high total sugar content of MJ.

The dispersibility of the MJ powders was over 97.7% and no significant differences among MJ powders were found. The high dispersibility indicates a low tendency to form lumps when MJ powders are added to water, facilitating their reconstitution. Furthermore, it is important to notice that the addition of the MJ powders did not modify the pH of the water.

**Figure 1:**
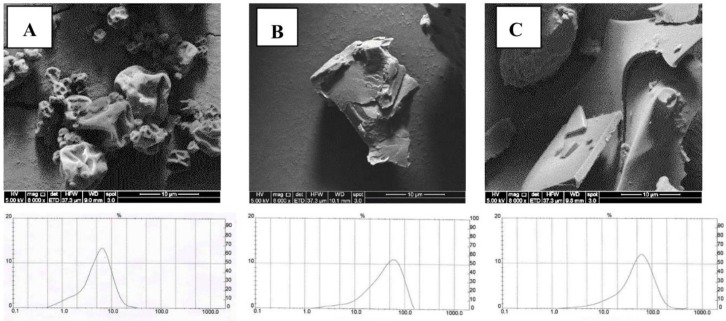
Scanning electron microscopic (SEM) photographs for SD (**A**), FDc (**B**) and FDm (**C**) powders and the distribution of particles diameter using Mastersizer.

Scanning electron microscopic photographs showed that SD powders (Figure 1A) have spherical shapes and particles with indented surfaces, obviating their agglomerating tendency. For FDc (Figure 1B) and FDm (Figure 1C) powders, SEM photographs showed undefined forms with more intended surfaces than SD powders, in agreement with the description by Fang and Bhandari [19] who suggested that the encapsulation method as well as the grinding process directly affects both the shape and size of powders.

The particle size measurement assumes powders have a spherical shape [20]. The particle size of SD powders was 6.4 µm, and 90% of the total sample had a diameter below 10.9 µm (Figure 1A) in line with the particle size of SD powders [16]. The particle size from FD powders (49.4 and 64.0 µm for FDc and FDm, respectively) was almost 10 times higher than the SD powder particles, and 90% of the total sample had a diameter below 93.9 and 117.1 µm, respectively. Therefore, in terms of the characteristics of MJ powders, the most relevant differences between SD and FD powders were the morphology and particle size that affect their solubility in water. However, the differences in particle size between FD powders did not have any impact on their solubility. MD is a water soluble biopolymer but SPI is partially soluble in water. However, MD blend with SPI represents a new system with different properties with respect to each single biopolymer [21], which explains why the solubility of maqui-juice microparticles with MD blend with SPI is lower than MD.

### 2.3. Stability of MJ Powders in Yogurt

**Figure 2:**
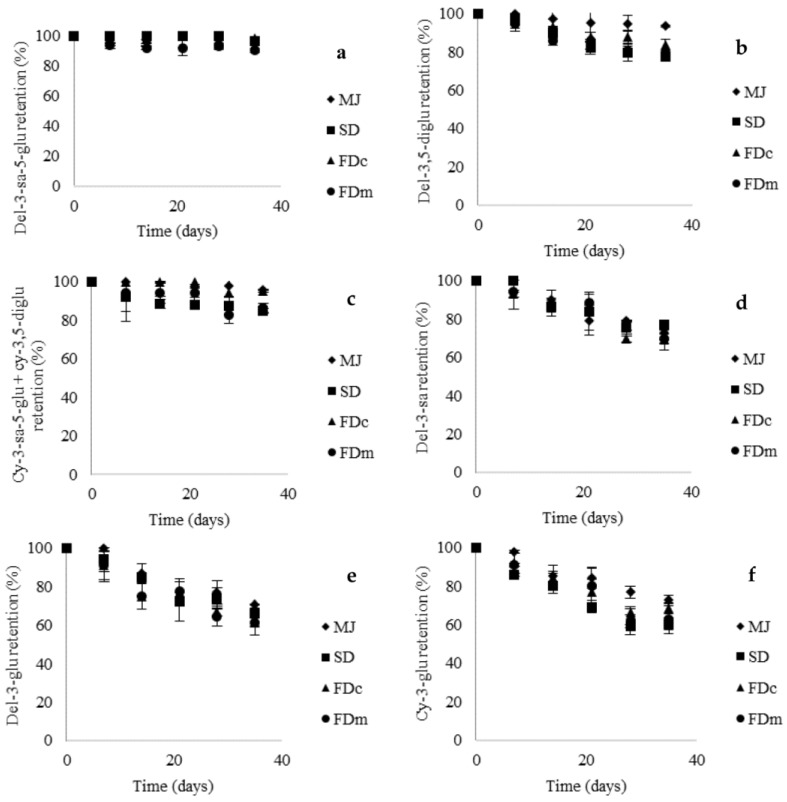
Evolution of the anthocyanins retention of MJ and MJ powders (SD, FDc and FDm) added to yogurt during storage at 5 °C. (**a**) delphinidin-3-sambubioside-5-glucoside retention; (**b**) delphinidin-3,5-diglucoside retention; (**c**) cyanidin-3-sambubioside-5-glucoside + cyanidin-3,5-diglucoside retention; (**d**) delphinidin-3-sambubioside retention; (**e**) delphinidin-3-glucoside retention; (**f**) cyanidin-3-glucoside retention.

Previous studies [10,22] on the evaluation of anthocyanin stability in yogurt has been focused on the retention of TA by spectrometry where the stability of anthocyanins comparing encapsulated with non-encapsulated anthocyanins has been scarcely studied [10]. The evolution of TA retention (Figure 2) was similar among MJ powders and MJ in agreement with results reported by Robert et al. [10] for anthocyanins from pomegranate juice and pomegranate microparticles. Furthermore, our results showed high retention of TA (SD, 82%, FDc, 88%, and FDm, 81%) after 35 days in line with a study carried out with a freeze-dried mulberry fruit juice [23]. Contrary Coisson et al. [22] and Robert et al. [10] reported that anthocyanins from acai and pomegranate juices, disappeared after 2 and 7 days of storage at 4 °C, respectively. This may be explained by the presence of different types of anthocyanins in fruits justifying the study of the stability of specific anthocyanins in yogurt.

Ribeiro et al. [24] indicated that in aqueous solutions, anthocyanins undergo structural rearrangements in response to changes in pH. Anthocyanins are most stable in acidic solutions (pH 1–3), whereas at a pH above 4, anthocyanins undergo chemical degradations to produce phenolic acids. However, as shown in Figure 2, the anthocyanins did not react uniformly; del-3-sa-5-glu showed the highest retention (90–99%) whereas cy-3-glu showed the lowest retention (61–69%) during storage at 5 °C after 35 days. Because the addition of MJ and MJ microparticles into yogurt did not modify the pH of the food matrix (4.3), anthocyanin degradation may be mainly attributed to the pH of the food matrix. Structural features of the anthocyanins influenced their stability in yogurt as reflected in their half-life values (Table 5). In this context, the *k_obs_* and the corresponding half-life values were calculated only for 3-*O*-monoglycosylated anthocyanins that reached a degradation of over 30%. The half-life of del-3-sa was significantly higher than del-3-glu and cy-3-glu, suggesting that the extra xylose as a sugar component of del-3-sa favors its stability in yogurt. On the other hand, the encapsulation method did not affect the stability of 3-*O*-monoglycosylated anthocyanins as reflected in the half-life values. This suggests that the differences in particle size and morphology between SD and FD powders in this study did not affect the stability of anthocyanins as other research [8,19] have proposed. The results also showed that MJ powders protected anthocyanins during the shelf-life of yogurt, preserving their stability, suggesting that yogurt might be a good carrier for functionalization.

### 2.4. Bioaccessibility of MJ Powders after an In Vitro Digestion

The BA has been defined as the amount of compound (anthocyanins) that was released from the food matrix after digestion [25]. In this study, BA was calculated in order to quantify the inhibition of anthocyanin degradation, comparing encapsulated (MJ powders) with non-encapsulated MJ anthocyanins. BA of TA in MJ powders (44.1% for SD and 43.8% for FD powders) was significantly higher than in MJ (35.2%) (Table 3). Nevertheless, no significant differences in the BA of TA between SD and FD powders were found, suggesting that their physical differences would not have an effect on the stability of anthocyanins.

Lucas-González et al. [26] demonstrated that the stability of maqui anthocyanins from a lyophilized fruit was greatly affected in the intestinal phase during in vitro GI digestion. Therefore, in our study, the expected low BA of TA in the MJ can be attributed to the high degradation of anthocyanins in GI conditions. It is well known that anthocyanins are unstable at high pH, and the shift from the acidic pH (pH 2) of the stomach to the almost neutral pH in the duodenum (pH 6) may be responsible for their specific hydrolysis and/or degradation [25,26,27,28]. Mouth conditions were considered in this study, although Mosele et al. [28] indicated that anthocyanins suffer negligible modifications due to the short exposure time in the mouth, resulting in a marginal effect of the α-amylase.

When anthocyanins are encapsulated, they are more protected in the GI tract, as was shown in our study. Oidtmann et al. [29] indicated that in bilberry microparticles encapsulated with polysaccharides (pectin amide) or proteins (whey protein isolate), anthocyanins were not degraded under simulated gastric conditions despite the fact that they had a competitive release from the microparticles. The same study indicated that in intestinal conditions, anthocyanins were also released from the microparticles (after 30 min), with degradation occurring after their release [29]. From another point of view, Flores et al. [27] indicated that the enzymatic and pH conditions are highly extractive in the stomach, which may affect the integrity of microparticles, favoring the release of anthocyanins. Nevertheless, the mixture of MD and SPI as EA demonstrated a protective effect, resulting in the enhancement of BA of TA in both SD and FD powders. Thus, our results suggest that this high BA is due to lower exposure times to gut conditions because of the protective effect of MD and SPI.

In concordance with the results of TA, BA of specific anthocyanins from MJ powders was significantly higher than MJ (Table 3). Moreover, there were significant differences in the BA among anthocyanins, where cy-3-sa-5-glu and cy-3,5-diglu had the highest BA (78.5% for SD and 76.5% for FD) and del-3-glu had the lowest BA (20.8% for SD and 22.6% for FD), demonstrating differences in their stability after their release from MJ powders. In agreement with our results, Lila et al. [25] suggest that the fact that cy-3-sa-5-glu plus cy-3,5-diglu demonstrated the highest BA is because two cyanidins combined with two linked sugars generate a more stable anthocyanin structure.

Despite the fact that changes in pH are involved in the degradation of anthocyanins in the GI tract and significant differences between the stability of 3-*O*-monoglycosylated and 3,5-*O*-diglycosylated anthocyanins as in yogurt were found (Table 3), the anthocyanins did not show the same behavior after the in vitro digestion. This can be contributed to the more complex reactions that anthocyanins experience during the digestion process, where the action of different enzymes in combination with different temperatures and time of exposure are all involved.

In this study, the results showed that the BA of both MJ microparticles (44.1–43.8%) and MJ (35.2%) were higher than that described by Lila et al. [25] for a semi-purified maqui extract (4%). These differences may be explained by the additional protector effect that MJ, as a food matrix, gives to anthocyanins. McDougall et al. [30] suggested that polyphenols generate linkages with the food matrix during digestion that may protect more labile anthocyanins against degradation.

## 3. Materials and Methods

### 3.1. Raw Material and Encapsulating Agents

Organic concentrated (65 °Brix) MJ (Patagonol™—LE, Bayas del Sur, Purranque, Chile) was microencapsulated using a mixture of MD (Maltodextrin 1520 Prinal S.A., Santiago, Chile) and SPI (Protein HS, min. 88%, Prinal S.A.). The MJ had the following characteristics: moisture content (41.0 ± 0.03%), total soluble solids (660 ± 1 g/kg), pH (3.8 ± 0.03), titratable acidity (58 ± 1 g/kg), and total sugars (610 ± 8 g/kg). Commercial natural yogurt (Soprole^®^, Santiago, Chile) was purchased in a local market in Santiago, Chile.

### 3.2. Preparation of MJ Powders by SD

Powders were prepared using MD and SPI (2:1) in 100 g solutions as follows: 2.5 g of MJ was mixed with 2.6–10.73 g of MD, 1.3–5.3 g of SPI and distilled water. The MD-SPI mixture in distilled water was stirred for 12 h and then the MJ was added. Each preparation was homogenized at 11,000 rpm for 5 min using a Polytron PT 2100 (Kinematica A.G, Luzern, Switzerland). The resulting solutions were fed into a mini spray-dryer B290 (Buchi, Flawil, Switzerland). The spray-dryer was operated at an inlet air temperature ranging from 120 to 180 ± 1 °C. Air flow, rate of feeding, and atomization pressure were 600 L/h, 1 mL/min, and 0.14 MPa, respectively. The resulting powders were stored absent of light at −80 °C until analyzed.

The experiment was performed using an orthogonal central composite experimental design with 12 runs (4 factorial points, 4 axial points and 4 central points). Independent variables were the inlet air temperature (120–180 °C) ($x_{1}$) and the MJ/MD-SPI ratio (1:2–1:6) ($x_{2}$). Dependent variables were the encapsulation efficiency (EE) of anthocyanins, the recovery, and the yield. A response surface methodology (RSM) was applied using the desirability function where 1 represented the maximization of each variable. The data were fitted to a second-order regression model. All experiments were conducted randomly to avoid systematic bias. The linear, quadratic, and interaction effects of the independent variables on the response variables at a confidence level of 95% were considered (Statgraphics Centurion XV, Version 15.1.02, StatPoint, Inc., Warrenton, VA, USA).

### 3.3. Preparation of MJ Powders by FD

Solutions (100 g) of MJ with MD and SPI (2:1) were prepared using the optimal MJ/MD-SPI ratio obtained for SD powders. Each solution was frozen (−80 °C) for 48 h and then dried in a LABCONCO freeze drier (LABCONCO Corporation, Kansas City, MO, USA) under a pressure below 0.004 mBar, and a temperature of −53 ± 1.7 °C for 48–72 h. The dried product was grinded by two different procedures, a coffee grinder (MKM6003, Bosch, Stuttgart, Germany) (FDc) and a porcelain mortar and pestle (3.5-inch) (FDm). The resulting powders were stored absent of light at −80 °C until analyzed.

### 3.4. Characterization of the MJ Powders Obtained by SD and FD

#### 3.4.1. Total Anthocyanin Determination in MJ Powders

The coating material structure of the microparticles was completely destructed to determine the total anthocyanins. The MJ powders (200 mg) were dispersed in 2 mL of methanol: acetic acid: water (50:1:49 *v*/*v*/*v*). This dispersion was vortexed for 1 min, ultrasonicated for 20 min, centrifuged at 11,000 rpm for 8 min, and then the supernatant was filtered (0.22 μm PTFE membrane filters, VWR International, Atlanta, GA, USA) and injected into the HPLC instrument.

#### 3.4.2. Surface Anthocyanin Determination in MJ Powders

Surface anthocyanins are determined using solvents where anthocyanins are soluble and the EA are insoluble. The MJ powders (400 mg) were dispersed in 2 mL of methanol: acetic acid (99:1 *v*/*v*). The dispersion was softly vortexed at room temperature for 1 min and then filtered (0.22 μm PTFE membrane filters, VWR International) and injected into the HPLC instrument.

The yield, recovery, and EE of anthocyanins were calculated according to the following equations:

(2) $$\mathrm{Yield}=\frac{\mathrm{powder}\;\mathrm{after}\;\mathrm{spray}\;\mathrm{drying}\;\left(g\right)}{\mathrm{solids}\;\mathrm{in}\;\mathrm{the}\;\mathrm{feed}\;\mathrm{solution}\;\left(g\right)}\;\times \;100$$

(3) $$\mathrm{Recovery}=\frac{\mathrm{anthocyanins}\;\mathrm{in}\;\mathrm{the}\;\mathrm{powder}\;\left(\mathrm{mg}/g\right)}{\mathrm{anthocyanins}\;\mathrm{in}\;\mathrm{the}\;\mathrm{feed}\;\mathrm{solution}\;\left(\mathrm{mg}/g\right)}\;\times \;100$$

(4) $$\mathrm{EE}\;\mathrm{anthocyanins}=\frac{{\mathrm{total}\;\mathrm{anthocyanin}}_{i}\;\mathrm{in}\;\mathrm{the}\;\mathrm{powder}-{\mathrm{surface}\;\mathrm{anthocyanin}}_{i}\;\mathrm{in}\;\mathrm{the}\;\mathrm{powder}}{{\mathrm{total}\;\mathrm{anthocyanin}}_{i}\;\mathrm{in}\;\mathrm{the}\;\mathrm{powder}}\;\times \;100$$

where i corresponds to each anthocyanin.

#### 3.4.3. Determination of Anthocyanins by HPLC

The LC-MS-IT-TOF identification of the anthocyanins was reported in our previous study [11]. Anthocyanin quantification was conducted on a HPLC system equipped with a DAD (Flexar, Perkin Elmer, Buckinghamshire, UK) using a C18 column (5 µm × 4.6 mm i.d. × 25 cm, Symmetry, Waters, Ireland), with 5% formic acid in H_2_O (A) and 100% methanol (B) as mobile phases. The flow rate was constant at 1 mL/min. Solvent gradient was 10%, 15%, 20%, 25%, 30%, 60%, 10%, and 10% of solvent B at 0, 5, 15, 20, 25, 45, 47, and 60 min, respectively [4]. The absorption was measured at a wavelength of 520 nm. Specific anthocyanins were quantified using a cy-3-glu (Sigma-Aldrich, St. Louis, MO, USA) calibration curve (0.16–24.8 µg/mL; R^2^ = 0.9998). Quantification of total anthocyanins (TA) content corresponds to the sum of all of the anthocyanins peaks.

#### 3.4.4. Physical Analysis of MJ Powders and Reconstitution

The moisture content and the a_w_ of MJ powders were measured according to AOAC method 925.40 [31]. The hygroscopicity was determined by the method described by Cai and Corke [32]. Powder bulk density was determined by loading the powder into a graduated cylinder to 10 mL mark and weighing it [33]. The powder weight and volume were then used to calculate the bulk density, expressed as mass/volume. The solubility and dispersibility as parameters of powder reconstitution were measured following Laokuldilok and Kanha [8]. MJ powders (1 g) were dissolved in 10 mL of distilled water and continuously stirred for 30 min. The suspension was then transferred to a Falcon tube and centrifuged at 6000 rpm for 20 min. The supernatant was dried at 105 °C for 24 h. The dry weight of the soluble solid was measured and used to calculate the solubility as a percentage. For dispersibility, MJ powders (1 g) were added to 10 mL of distilled water and then stirred vigorously by a magnetic stirrer for 25 s. The reconstituted powder was passed through a 150 mm sieve. An aliquot (1 mL) of the sieved solution was dried at 105 °C for 4 h. After that, total solids as a percentage was used to calculate dispersibility.

#### 3.4.5. Powder Morphology

Scanning electron microscopy was used to study the outer structures of the MJ powders. The samples were coated with a 10 nm film of gold by a Sputter Coater 108auto with a thickness Controller MTM-20 (Cressington Scientific Instruments, Watford, UK) and analyzed with a High Resolution Scanning Electron Microscope (HR-SEM) with a secondary electron detector (SED) (model INSPECT-F50, FEI, Thermo Fisher Scientific, Hillsboro, OR, USA) operated at 5.00 KV.

#### 3.4.6. Particle Size

The size of the MJ powders (SD, FDc and FDm) was measured using a MastersizerX (Malvern Instruments, Worcestershire, UK). The samples were dispersed in isopropanol and sonicated for 20 s in enough powder to reach the optimal shade in the equipment. The mean particle size was expressed as the volume mean diameter (D_4,3_).

### 3.5. Stability of Anthocyanins from MJ Powders in Yogurt

MJ (SD, FDc and FDm) powders (0.25 g) were added to yogurt (25 g) in glass containers with tap and stored at 5 ± 1 °C in the absence of light for 5 weeks (in triplicate). A control sample (in triplicate) with the addition of MJ (0.25 g) was also prepared. The containers were withdrawn every 7 days to determine TA and specific anthocyanin retention [21]. Yogurt with MJ (5 g) and yogurt with MJ powders (7 g) were mixed with LiChrosolv^®^ chromatography (grade LC-MS, Merck-Milipore, Burlington, MA, USA) water (pH = 3) until reaching 10 g. Each sample was vortexed for 1 min, and centrifuged at 6,000 rpm for 20 min. The supernatant was centrifuged at 12,000 rpm for 20 min. Following previous studies, first-order degradation kinetic model (lnC = lnC_0_ − *k*(t)) were used to fit the analytical data and to calculate degradation rate constants (*k_obs_*) obtained from the slope of a plot of the natural log of the specific anthocyanin content vs. time. Retention (%) = (C_t_/C_0_) × 100, where C_t_ is the anthocyanin content at “t” time, and C_0_ is the initial specific anthocyanin content. Half-life values were calculated from: (t_1/2_ = Ln2/*k_obs_*) [10,21]*.*

### 3.6. Bioaccessibility of MJ Powders Using an In Vitro Digestion Model

Samples of 0.5 g (in triplicate) of MJ powders and MJ were processed following Aravena et al. [34].

#### 3.6.1. Mouth Digestion

Artificial saliva (9 mL) was added to each flask with sample. This solution was comprised of 14.4 mM sodium bicarbonate, 21.1 mM potassium chloride, 1.59 mM calcium chloride, and 0.2 mM magnesium chloride. The pH was adjusted to 7 with HCl (1 M). Sixty α-amylase units per milliliter of buffer were incorporated the same day the test was performed. Samples were incubated in a thermostatic bath (ZHWY-110X30, Zhicheng, Shangai, China); 37 °C for 5 min, at a shaking speed of 185 rpm.

#### 3.6.2. Stomach Digestion

The pH of the samples was adjusted to 2.0 using HCl (1 M), then 36 mL of a pepsin solution (25 mg/mL in 0.02 M HCl) was added. Therefore, each sample (containing 9 mL of artificial saliva) was diluted 5-fold with artificial gastric juice, as it occurs in the stomach. Samples were incubated for 2 h at 37 °C with a stirring speed of 130 rpm.

#### 3.6.3. Gut Digestion

The pH of the samples was adjusted to 6.0 with NaHCO_3_ (1 M). Then, 0.25 mL per mL of sample of an artificial gut solution, containing pancreatin (2 g/L) and bile salts (12 g/L) dissolved in aqueous NaHCO_3_ (0.1 M) was added. Incubation was carried out for 2 h at 37 °C and a shaking speed of 45 rpm.

Each digestion product was transferred to 50 mL Falcon tubes and the pH was adjusted (pH 3) [27]. It was then centrifuged for 10 min at 5000 rpm to recover the liquid fraction. Next, the liquid digestion product was centrifuged at 12,000 rpm before anthocyanin analysis. The measured amount of specific anthocyanin was the bioaccessible portion, which was calculated as follows:

(5) $$\mathrm{Bioaccessibility}\;\%=\frac{{\mathrm{mg}\;\mathrm{anthocyanin}}_{i}\;\mathrm{on}\;\mathrm{digest}\;\mathrm{product}}{{\mathrm{mg}\;\mathrm{anthocyanin}}_{i}\;\mathrm{in}\;\mathrm{MJ}\;\mathrm{or}\;\mathrm{MJ}\;\mathrm{powders}}\;\times \;100$$

where i corresponds to each anthocyanin quantified.

### 3.7. Statistical Analysis

The differences in MJ powders for each parameter and among specific anthocyanins were analyzed using a one-way ANOVA test for means comparison. When significant differences were found, the Tukey HSD (honest significant differences) multiple-comparison test (*p* ≤ 0.05) was applied. Analyses were performed with SAS 9.2 for Microsoft Windows (2009; SAS Institute Inc., Cary, NC, USA).

## 4. Conclusions

The studied encapsulation methods did not generate differences on stability of 3-*O*-monoglycosylated anthocyanins in yogurt, and either on BA of anthocyanins after in vitro GI digestion. Thus the encapsulation technology using either SD or FD can be used as a protection strategy for MJ anthocyanins by producing stable anthocyanin-rich powders to be used in yogurt, also improving their BA by 9%. These results contribute to the development of new food health ingredients using maqui anthocyanins.

## Figures and Tables

**Table 1:** Orthogonal central composite design showing factor’s level combinations and results for encapsulation of MJ anthocyanins by SD.

Run	Inlet Air Temperature (°C)	MJ/EA Ratio	Yield (%)	Recovery (%)	EE (%)						
					TA	Del-3-sa-5-glu	Del-3,5-diglu	Cy-3-sa-5-glu+Cy-3,5-diglu	Del-3-sa	Del-3-glu	Cy-3-glu
1	120	1:2	76.3	88.1	77.7	84.0	79.4	81.1	79.9	77.1	73.4
2	180	1:2	74.6	93.3	78.8	83.1	79.0	80.6	79.0	77.1	74.2
3	120	1:6	69.1	90.3	91.9	93.5	92.7	91.7	90.3	89.3	88.8
4	180	1:6	72.1	93.3	89.4	90.9	88.8	88.1	87.2	84.6	84.6
5	114	1:4	73.7	91.4	90.7	92.6	91.3	90.6	87.8	87.7	87.4
6	186	1:4	73.9	96.1	90.0	90.4	88.9	89.1	87.8	86.4	86.1
7	150	1:1.6	74.8	100.0	74.7	76.6	71.1	71.5	71.5	66.5	65.3
8	150	1:6.4	70.5	82.6	91.6	92.2	90.9	90.8	88.9	87.7	87.4
9	150	1:4	71.0	91.8	89.7	91.0	88.8	88.8	86.9	83.8	84.7
10	150	1:4	69.4	90.1	87.3	89.5	87.6	86.6	85.1	82.2	83.2
11	150	1:4	71.5	94.0	89.3	90.6	89.3	88.6	86.6	84.8	86.1
12	150	1:4	69.5	90.1	90.1	90.9	89.7	89.6	86.0	85.7	85.0

TA = total anthocyanins; del-3-sa-5-glu = delphinidin-3-sambubioside-5-glucoside; del-3,5-diglu = delphinidin-3,5-diglucoside; cy-3-sa-5-glu+cy-3,5-diglu = cyanidin-3-sambubioside-5-glucoside+cyanidin-3,5-diglucoside; del-3-sa 5 = delphinidin-3-sambubioside; del-3-glu = delphinidin-3-glucoside; cy-3-sa = cyanidin-3-sambubioside; cy-3-glu = cyanidin-3-glucoside.

**Table 2:** Regression coefficients of fitted equations for each response variables.

	$y_{n}$	$\beta_{n0}$	$\beta_{n1}$	$\beta_{n2}$	$\beta_{n3}$	$\beta_{n4}$	$\beta_{n5}$	R^2^	R^2^ (Adjusted for d.f.)
	Yield (%)	136.3140	−0.6917	−6.1670	0.0021	0.0196	0.2692	0.902	0.820
	Recovery (%)	100.6240	−0.1919	1.5889	0.0010	−0.0092	−0.1968	0.429	0.00
EE%	TA	57.4578	−0.0461	14.7790	0.0003	−0.0150	−1.1579	0.979	0.961
	del-3-sa-5-glu	90.3751	−0.3418	11.3291	0.0011	−0.0071	−0.9569	0.963	0.933
	del-3,5-diglu	78.7725	−0.3441	15.8078	0.0012	−0.0146	−1.2780	0.972	0.949
	cy-3-sa-5-glu+cy-3,5-diglu	88.4993	−0.4300	14.0942	0.0015	−0.0129	−1.1456	0.930	0.872
	del-3-glu	94.0431	−0.4748	11.6651	0.0016	−0.0092	−0.9285	0.951	0.909
	cy-3-sa	102.4870	−0.7099	15.2545	0.0025	−0.0196	−1.1305	0.927	0.866
	cy-3-glu	70.4168	−0.3790	18.4930	0.0015	−0.0208	−1.4470	0.971	0.947

TA = total anthocyanins; del-3-sa-5-glu = delphinidin-3-sambubioside-5-glucoside; del-3,5-diglu = delphinidin-3,5-diglucoside; cy-3-sa-5-glu+cy-3,5-diglu = cyanidin-3-sambubioside-5-glucoside+cyanidin-3,5-diglucoside; del-3-sa 5 = delphinidin-3-sambubioside; del-3-glu = delphinidin-3-glucoside; cy-3-sa = cyanidin-3-sambubioside; cy-3-glu = cyanidin-3-glucoside.

**Table 3:** Anthocyanins content in MJ and MJ powders and the BA of MJ anthocyanins after an in vitro digestion model.

	MJ		SD		FD	
	mg/g	BA %	mg/g	BA %	mg/g	BA %
Del-3-sa-5-glu	7.4 ± 0.02	35.1 ± 3.35 ^b,A^	1.3 ± 0.04	43.2 ± 0.61 ^c,AB^	1.4 ± 0.07	40.8 ± 2.00 ^cd,B^
Del-3,5-diglu	3.3 ± 0.01	36.4 ± 1.12 ^b,A^	0.6 ± 0.02	34.6 ± 1.16 ^b,A^	0.6 ± 0.03	36.2 ± 1.9 ^c,A^
Cy-3-sa-5-glu + cy-3,5 diglu	3.4 ± 0.01	48.9 ± 1.58 ^c,A^	0.7 ± 0.01	73.5 ± 3.04 ^d,B^	0.6 ± 0.03	76.5 ± 2.39 ^e,B^
Del-3-sa	1.5 ± 0.02	21.9 ± 2.83 ª^,A^	0.3 ± 0.01	24.8 ± 0.85 ^a,AB^	0.3 ± 0.01	28.5 ± 2.56 ^b,B^
Del-3-glu	2.4 ± 0.01	21.2 ± 0.50 ª^,A^	0.4 ± 0.01	20.8 ± 0.41 ^a,A^	0.4 ± 0.02	22.6 ± 0.47 ^a,B^
Cy-3-glu	1.2 ± 0.02	24.0 ± 2.29 ª^,A^	0.2 ± 0.01	43.8 ± 2.93 ^c,B^	0.2 ± 0.01	44.8 ± 1.00 ^d,B^
3-*O*-monoglycosylated anthocyanins	5.0 ± 0.01	23.3 ± 3.29 ^a,A^	0.9 ± 0.02	27.2 ± 0.57 ^a,AB^	0.9 ± 0.04	30.8 ± 2.09 ^a,B^
3,5-*O*-diglycosylated anthocyanins	14.1 ± 0.04	39.5 ± 3.18 ^b,A^	2.6 ± 0.06	50.1 ± 2.07 ^b,B^	2.6 ± 0.12	48.4 ± 0.90 ^b,B^
TA HPLC	17.5 ± 0.04	35.2 ± 3.21 ^A^	3.3 ± 0.20	44.1 ± 1.68 ^B^	3.3 ± 0.41	43.8 ± 0.13 ^B^

Mean values (*n* = 3) and standard deviation that are followed by different upper case letters in the same row indicate significant differences (*p* ≤ 0.05) between encapsulation methods and by different lower case letters in the same column indicate significant differences (*p* ≤ 0.05) among anthocyanins. MJ, maqui juice; SD, powders obtained under spray-drying optimal conditions; FD, powders obtained by freeze-drying grinded product; TA, total anthocyanins; BA, bioaccessibility. del-3-sa-5-glu, delphinidin-3-sambubioside-5-glucoside; del-3,5-diglu, delphinidin-3,5-diglucoside; cy-3-sa-5-glu, cyanidin-3-sambubioside-5-glucoside; cy-3,5-diglu, cyanidin-3,5-diglucoside; del-3-sa, delphinidin-3-sambubioside; del-3-glu, delphinidin-3-glucoside; cy-3-glu, cyanidin-3-glucoside.

**Table 4:** MJ powders characteristics and parameters of powders reconstitution.

MJ Powder	SD	FDc	Fdm
EE TA	92.5 ± 0.02 ^a^	93.0 ± 0.01 ^a^	93.9 ± 0.01 ^a^
EE 3-*O*-monoglycosylated anthocyanins	89.5 ± 2.2 ^a,A^	91.4 ± 1.3 ^ab,A^	92.3 ± 1.4 ^b,A^
EE 3,5-*O*-diglycosylated anthocyanins	93.1 ± 1.4 ^a,B^	94.6 ± 0.6 ^ab,B^	95.4 ± 0.9 ^b,B^
Recovery (%)	99.8 ± 0.01 ^b^	91.9 ± 0.01 ^a^	91.8 ± 0.02 ^a^
Yield (%)	64.1 ± 0.01 ^a^	94.6 ± 0.01 ^b^	94.6 ± 0.01 ^b^
a_w_	0.3 ± 0.01 ^b^	0.1 ± 0.01 ^a^	0.1 ± 0.01 ^a^
Moisture content (%)	6.4 ± 0.02 ^b^	3.2 ± 0.01 ^a^	3.2 ± 0.02 ^a^
Hygroscopicity (%)	39.4 ± 0.03 ^a^	48.0 ± 0.02 ^b^	53.1 ± 0.01 ^b^
Bulk density (g/mL)	0.4 ± 0.01 ^b^	0.3 ± 0.01 ^a^	0.3 ± 0.01 ^a^
Solubility (%)	70.4 ± 0.01 ^b^	59.9 ± 0.02 ^a^	59.1 ± 0.02 ^a^
Dispersibility (%)	99.1 ± 0.01 ^a^	100.0 ± 0.01 ^a^	97.7 ± 0.01 ^a^
pH	5.3 ± 0.02 ^b^	5.2 ± 0.01 ^a^	5.2 ± 0.02 ^a^

Mean values (*n* = 3) and standard deviation that are followed by different lower case letters in the same row indicate significant differences (*p* ≤ 0.05) between encapsulation methods and by different upper case letters in the same column indicate significant differences (*p* ≤ 0.05) among anthocyanins. EE, encapsulation efficiency; TA, total anthocyanins; SD, powders obtained under spray-drying optimal conditions; FDc, powders obtained by freeze-drying grinded with coffee grinder; FDm, powders obtained by freeze-drying grinded with porcelain mortar and pestle.

**Table 5:** Half-life (t½) values (days) of anthocyanins and anthocyanins retention (time day 35, t_35_) from MJ and MJ powders in yogurt during storage at 5 °C.

	MJ (Control)		SD		FDc		FDm	
	t_1/2_ (day)	Retention t_35_ (%)	t_1/2_ (day)	Retention t_35_ (%)	t_1/2_ (day)	Retention t_35_ (%)	t_1/2_ (day)	Retention t_35_ (%)
Del-3-sa-5-glu	nd	100 ± 0.02	nd	97 ± 0.01	nd	99 ± 0.01	nd	90 ± 0.02
Del-3,5-diglu	nd	94 ± 0.02	nd	78 ± 0.02	nd	84 ± 0.03	nd	79 ± 0.03
Cy-3-sa-5-glu+cy-3,5-diglu	nd	96 ± 0.03	nd	85 ± 0.01	nd	95 ± 0.01	nd	86 ± 0.01
Del-3-sa	75 ± 8 ^b,A^	73 ± 0.03	75 ± 11 ^b,A^	77 ± 0.01	67 ± 11 ^b,A^	69 ± 0.02	70 ± 4 ^b,A^	70 ± 0.02
Del-3-glu	65 ± 9 ^a,B^	71 ± 0.02	59 ± 5 ^ab,AB^	66 ± 0.02	48 ± 8 ^a,A^	62 ± 0.01	46 ± 4 ^a,A^	62 ± 0.01
Cy-3-glu	68 ± 7 ^a,C^	73 ± 0.01	44 ± 2 ^a,A^	61 ± 0.02	59 ± 9 ^a,BC^	69 ± 0.02	50 ± 7 ^a,B^	63 ± 0.02

Mean values (*n* = 3) and standard deviation that are followed by different upper case letters in the same row indicate significant differences (*p* ≤ 0.05) between encapsulation methods and by different lower case letters in the same column indicate significant differences (*p* ≤ 0.05) among anthocyanins. del-3-sa-5-glu, delphinidin-3-sambubioside-5-glucoside; del-3,5-diglu, delphinidin-3,5-diglucoside; cy-3-sa-5-glu, cyanidin-3-sambubioside-5-glucoside; cy-3,5-diglu, cyanidin-3,5-diglucoside; del-3-sa, delphinidin-3-sambubioside; del-3-glu, delphinidin-3-glucoside; cy-3-glu cyanidin-3-glucoside; SD, powders obtained under spray-drying optimal conditions; FDc, powders obtained by freeze-drying grinded with coffee grinder; FDm, powders obtained by freeze-drying grinded with porcelain mortar and pestle; nd, not determined.

## Abbreviations

a_w_	water activity
BA	bioaccessibility
cy-3-glu	cyanidin-3-glucoside
cy-3-sa	cyanidin-3-sambubioside
cy-3-sa-5-glu	cyanidin-3-sambubioside-5-glucoside
cy-3,5-diglu	cyanidin-3,5-diglucoside
del-3-sa-5-glu	delphinidin-3-sambubioside-5-glucoside
del-3,5-diglu	delphinidin-3,5-diglucoside
del-3-glu	delphinidin-3-glucoside
del-3-sa	delphinidin-3-sambubioside
FD	freeze-drying
GI	gastrointestinal
EA	encapsulating agents
EE	encapsulation efficiency
HPLC-DAD	high-performance liquid chromatography–photo diode array detector
MD	maltodextrin
MJ	maqui juice
RSM	response surface methodology
SD	spray-drying
SPI	soy protein isolate
TA	total anthocyanins
SEM	scanning electron microscopy
